# Intercultural communicative competence among Indonesian migrant workers in Malaysia: a qualitative exploration

**DOI:** 10.3389/fsoc.2024.1321451

**Published:** 2024-03-27

**Authors:** Djatmika, Bahtiar Mohamad, Riyadi Santosa, Agus Hari Wibowo

**Affiliations:** ^1^Faculty of Cultural Science, Universitas Sebelas Maret, Surakarta, Indonesia; ^2^Othman Yeop Abdullah Graduate School of Business (OYAGSB), Universiti Utara Malaysia, Kuala Lumpur, Malaysia

**Keywords:** migrant workers, intercultural communication, intercultural communicative competence, labor market integration, Indonesia, migration, labour market, global mobility

## Abstract

Millions of Indonesian migrant workers have sought employment in Malaysia during the last three decades. Many of them are skilled and unskilled laborers, and their incorporation into the host society’s labor market has the potential to improve their own quality of life while also contributing significantly to the country’s economy. However, Indonesian migrant workers encounter numerous problems in their professional and personal lives. Therefore, this study aims to investigate the intercultural communicative competence (ICC) factors as one of the Indonesian migrant workers’ strategies. This is qualitative exploratory research on the factors of ICC in the integration of Indonesian migrant workers into the Malaysian labor market. Focus groups were used to obtain data from 16 Indonesian migrant workers who had already successfully integrated into the Malaysian workforce as well as newcomers who were in the process of integrating into the local culture. In addition, interviews have been conducted with the 13 employers to complement the data from the migrant workers. The data was analyzed using rounds of deductive and inductive coding and analysis based on the five components of Byram’s model. The findings suggest that practicing intercultural communication skills can help migrant employees overcome cultural difficulties in the Malaysian labor market. The Indonesian migrant workers and their employers also indicated that they have an advantage in cultural integration due to the cultural similarities. The paper discusses the implications of the findings in terms of ICC training offered to migrant workers themselves (culture and language) and to professionals who work with them, with the goal of facilitating and promoting Indonesian migrant workers’ labor market integration.

## 1 Introduction

With a population of 273.8 million (2021), it is estimated that around 4.5 million Indonesians work abroad. Indonesian migration to other countries has been a significant phenomenon for several decades, driven by various factors such as economic opportunities, labor demand, and political circumstances. Malaysia has been a major destination for Indonesian migrant workers. The proximity between the two countries, shared cultural ties, and labor demand in sectors such as construction, plantation, and manufacturing have contributed to this migration flow. As of 2022, based on Bank Indonesia data, it is estimated that over 1.6 million Indonesian migrants reside in Malaysia. Other countries, such as Saudi Arabia, Singapore, Hong Kong, Taiwan, and other Middle East and Gulf countries, have been popular destinations for Indonesian migrant workers, particularly in the domestic work sector. As of 2022, it is estimated that there are approximately 833,810 Indonesian migrants in Saudi Arabia, 294,460 in Taiwan, 290,430 in Hong Kong, 92,310 in Singapore, and several hundred thousand in Middle East and Gulf countries (Bank Indonesia, 2022).

Economic motivations drive people to be extremely competitive in seeking employment in Indonesia, and others will choose to work abroad. The enormous number of Indonesians who opt to work in other countries will undoubtedly have an impact on the country’s income and economic growth (Fathoni et al., 2017). Economic considerations can be detected sociologically as key determinants of geographical migration, for example, by keeping track of the growing number of international migrant workers. However, it should be noted that economic incentives are not the main reason for migration, although they are a major influence in many cases. Migration reasons can be divided into two categories: centrifugal and centripetal forces. Centrifugal causes such as bad environmental conditions, limited geographical accessibility, limited living facilities, and economic possibilities all lead migrants to leave their home nations (Ariadi et al., 2019). Centripetal influences draw migrants to specific areas that are often thought to be better environmentally, socioculturally, and economically than their native countries. Malaysia possesses centripetal influences that have drawn numerous Indonesian migrant workers, particularly the similarities in sociocultural terms between the two countries. The two countries have land and sea boundaries, the same predominant religion (Islam), and linguistic and cultural connections (Djafar and Hassan, 2012).

Economic factors are perhaps the most widely mentioned cause of migration, while cultural, social, and political issues are less prominent (Todaro, 1979). Mantra and Davies (1989) contend that more constrained economic conditions in the origin country motivate individuals to relocate to countries with better economic opportunities. Thus, migration occurs when there is a utility value differential between two or more areas, and the destination has a higher utility value than the country of origin (Haris, 2002). In line with this, Goma (1993) asserts that the disparity in economic conditions across different parts of Indonesia is the fundamental cause of large-scale migration. Working in specific destinations is thought to be more profitable than working in one’s home country.

Aside from economic challenges, migrant workers face new circumstances in their lives that are vastly different from those in their native countries. Many studies have demonstrated that cultural variations are a major factor. For example, workplace diversity has highlighted the critical importance of a networked society. Nowhere is this need more acute than in the midst of a worldwide epidemic, which has prompted individuals to look for new methods to “live together” and feel connected (Deardorff, 2015, 2020). This realization has highlighted the increased need to understand what it means to effectively communicate across and between cultures (Adamu and Mohamad, 2019). Individually, an increasing number of people have identities and opinions shaped by their interactions with diverse people.

Global mobility has produced a societal climate in which people must learn about “good” communication with individuals who do not share their values and beliefs. It brought about a societal landscape where individuals must grapple with the complexities of communicating effectively with those who hold differing values and beliefs (Hofstede, 2001; Inglehart and Norris, 2016). As people increasingly traverse borders for work, education, and leisure, encounters with individuals from diverse cultural backgrounds have become commonplace (Inglehart and Norris, 2016). This reality underscores the importance of developing intercultural communication competence, which involves the ability to understand, respect, and adapt to cultural differences (Bennett, 1993; Lustig and Koester, 2010). As a result, establishing a sense of “oneness” is critical, in which people must learn to connect positively with people who speak different languages and hold different values. This is a strong reminder that establishing positive cross-cultural connections requires intercultural competency, which is no longer a choice. Arasaratnam (2015) stated in an assessment of her 10-year studies in intercultural competence that “competence” must be studied on an ongoing basis.

Given this, further research is needed to better understand ICC and related concerns among Indonesian migrant workers, especially in Malaysia, where the culture is made up of a variety of ethnic and racial components (Bakar et al., 2016; Raza et al., 2018). A review of the literature identifies three major concerns about migrant workers’ ICC in Malaysia. First, there is the issue of host-culture people having unrealistic expectations of migrant employees. For example, while migrant workers are important to Malaysia’s economic success, their presence in a culture that is distinct from others can result in a number of societal concerns. Migrant workers form their own separate group since they contribute their own cultural values, traditions, and beliefs to Malaysian society, and their presence has raised anxiety among Malaysians (Lasimbang et al., 2016; Aziz et al., 2017; Merall, 2018; Mohamad et al., 2018). The conflicts that come with developing multicultural communities are clear, as it is difficult for culturally diverse groups to live, work, play, and communicate harmoniously together (Lustig and Koester, 2010). Second, migrant workers suffer significant obstacles as a result of racism and prejudice by members of the local society. The migrant workers do not start on the same level playing field as the rest of the population (Hebbani and Khawaja, 2019). Therefore, failure to foster communal harmony among culturally different individuals can have severe effects on the nation. As a result, “the other” (i.e., migrant workers) must learn to coexist peacefully with fellow citizens.

Third, according to Malaysian government estimates, at least two million migrant workers, largely from Indonesia and Bangladesh, make about 15% of the overall working population (Ministry of Home Affairs, 2019; Department of Statistics Malaysia Official Portal, 2021). The increasing number of migrant workers produces a lot of problems, including unacceptable behavior in local culture (Sakolnakorn, 2019), bad societal perceptions (Merall, 2018), and weak language and communication skills (Lasimbang et al., 2016). As a result, assessing ICC from the perspective of migrants is crucial in order to aid them in minimizing cultural differences and engaging effectively in society.

Based on the three major challenges, it is clear that the presence of migrant workers, particularly Indonesian labor, has influenced Malaysian society. Despite this, little is known about this community due to a lack of ICC research, particularly on migrant workers in Malaysia. Previous research has generated descriptions of skills, characteristics, and behaviors that aid in understanding ICC. However, much of the research on ICC in expatriates (mainly diplomats and business people) or sojourners (particularly international academic/student migrants) has been conducted (Spitzberg, 2000, 2012; Lustig and Koester, 2010; Deardorff, 2011). As a result, academics must investigate the ICC of migrant workers from specific countries, such as Indonesia. With the deficiencies of a better recognized understanding of what ICC comprises, the purpose of this article is to investigate the ICC factors as one of the migrant workers’ strategies.

This paper begins by discussing the essential elements that comprise ICC before explaining the methodology of the study to offer the necessary theoretical backdrop. Then, ICC among Indonesian migrant workers who had previously successfully integrated into Malaysian labor markets was examined. The findings are discussed in terms of the function of ICC in this process as well as the potential implications for interventions aimed at assisting migrant workers’ integration into the host society’s labor market, with the caveat that the findings are extremely preliminary. The discussion focused not only on ICC-focused initiatives with Indonesian migrant workers but also on their employers, who frequently engage with them.

## 2 Literature review

### 2.1 The conceptualization of intercultural communication competence

Deardorff (2004) defines intercultural communication competence (ICC) as the ability to communicate effectively and appropriately in multicultural circumstances based on one’s intercultural knowledge, skills, and attitudes. The possession and comprehension of resources that inform the performance of abilities in a specific circumstance, including the ability to acquire informational resources through questioning, observation, cognitive modeling, or creative reflection, is referred to as knowledge. Skills are goal-directed, repetitive behavioral sequences that result in some amount of goal attainment. Given the fundamental components of intercultural competence, it is hypothesized that the more a person knows, feels motivated, and engages in skilled behaviors, the greater the likelihood that the individual will be seen as skilled in intercultural communication (Deardorff, 2006; Byram and Golubeva, 2020).

Moreover, the essential notion of ICC is founded on the concepts of appropriateness and efficacy. Appropriateness is concerned with avoiding violations of social or interpersonal norms, regulations, or expectations, whereas effectiveness is inextricably related to the satisfaction of achieving intended goals (Spitzberg and Cupach, 1984, 1989). Affective communication necessitates a grasp of the standards that apply in a given situation (Lustig and Koester, 2010). Knowing what behavior is appropriate and what is not in a specific setting allows one to act in a way that either adheres to or violates the norms. Intercultural competence models discovered that motivation, knowledge, and abilities that compose intercultural competence, as well as the concepts of appropriateness and effectiveness, are the most frequently used elements (Spitzberg and Changnon, 2009). Scholars, on the other hand, do not always use these terminologies precisely to describe their models. For example, motivation is related to the various positive and negative valences that push communication in one direction or another (Spitzberg, 2012). Knowledge is defined as an understanding of resources that support the use of talents in a certain scenario. This entails being able to gather information through inquiries, observations, cognitive modelling, or imaginative introspection.

Skills are repeating, goal-directed, behavior-based patterns that result in some level of goal achievement. Given the main components of intercultural competence, it is hypothesized that the greater one’s knowledge, motivation, and skilled conduct, the greater one’s likelihood of being considered competent in intercultural communication (Deardorff, 2006; Byram and Golubeva, 2020). Early attempts to assess ICC relied mainly on an individual’s attributes (personal characteristics) that predispose them to perform well in a foreign culture (Ruben, 1989). This focus could be traced back to the beginnings of the field of intercultural communication. Following WWII, the United States attempted to invest in and engage in foreign nations, and numerous individuals were dispatched abroad to carry out global missions (Moon, 1996). However, due to their inability to adapt to cultural differences, expats failed to accomplish their tasks and returned. Cross-cultural effectiveness, personal adaptability, and culture shock are also problems for sojourners. The requirement for workers to be trained to operate effectively in a foreign environment sparked scientific interest in the concept of ICC. Ruben (1989) observed that early intercultural communication studies required ICC perspectives to achieve three goals: to explain failures and anticipate success abroad, to develop methods for staff selection, and to evaluate sojourner training and preparation procedures.

The competency in the literature is still strongly based on personality attributes and is almost always tested as such (Spitzberg and Changnon, 2009). For instance, the trait-like approach posits that communicators can act appropriately in a variety of communication circumstances (Lustig and Koester, 2010). Trait orientation employs personality-based theory to explain competence. Personality qualities associated with competence include self-involvement, interpersonal sensitivity, openness, mindfulness, empathy, and resourcefulness. People are competent because they are empathic, good listeners, and forceful, according to their trait orientation. Since then, studies on the topic have increased, emphasizing a variety of contexts (international student roommates, language education, business, study abroad, higher education, overseas development projects, and so on) and applying a variety of approaches with a range of nomenclature that includes, among other things, appropriateness, effectiveness, and competence (Martin and Nakayama, 2013; Sabet and Chapman, 2023).

In terms of intercultural competence research, Hammer’s (2015) review of intercultural competence research focuses on personal qualities. As a result, the cognitive, emotional, and behavioral (CAB) dimensions, which are fundamentally compositional, were used to investigate distinct personal qualities. Individual factors, such as ambiguity tolerance, open-mindedness, and behavioral adaptation, are highlighted as components of intercultural competence in this approach. Hammer (2015) defines intercultural competence as self-confidence, expectations, initiative, intercultural skills, stress management, nonverbal behaviors, cultural awareness, and a cross-cultural mindset. Hammer (2015) presented a developmental paradigm as an alternate strategy for dealing with the overlap and large list of elements for intercultural competence. The developmental model of international sensitivity, for example, studies how people move from a low to a high level of international competence. In this way, it depicts how individuals progress from a basic to a more comprehensive understanding of intercultural competence (Bennett, 1986; Lee et al., 2023).

There is a wide variety of methodologies, theoretical assumptions, and individual-level variables in the literature that may affect ICC directly or indirectly (see Arasaratnam, 2007; Arasaratnam, 2016). At this point, it is crucial to call attention to the most popular ways used by academics to address ICC by using the general culture approach, which found several elements that might directly or indirectly influence the ICC of people from different cultures. Given its universal application, the general cultural approach to ICC has evolved immensely in the field of ICC (i.e., Byram, 1997).

While considerable ICC research has been conducted in numerous contexts around the world, there is a scarcity of studies addressing the role of ICC in migrant workers’ integration in their host countries, particularly their labor market integration. This study’s overarching purpose was to assess the ICC of Indonesian migrant workers in order to improve their employability and social integration. While the study is exploratory in nature, it contributes significantly to our understanding by focusing on migrant workers who have already successfully integrated into the host society’s labor market (working in Malaysia for more than 5 years). The research question we intend to address is: Does the ICC of Indonesian migrant workers influence how they deal with the obstacles and barriers they confront in the host society’s labor market?

### 2.2 Indonesian migrant workers in Malaysia

Indonesian migrant workers in Malaysia have a long history that has been shaped by numerous economic, political, and social circumstances. The Malaysian government divides migrant workers into two categories: foreign employees and expatriates. Foreign workers are individuals with a monthly pay of less than RM5,000, while expatriates have a larger salary (Kanapathy, 2008). The influx of Indonesian migrants’ workers to Malaysia may be traced back to the 1970s, a period of significant economic growth and modernization in Malaysia, especially in the construction, plantation, agriculture, and manufacturing industries. Therefore, Indonesia and Malaysia signed a Memorandum of Understanding (MoU) in the early 1980s to restrict labor mobility between the two nations to protect the rights and welfare of Indonesian employees, provide recruitment procedures, and handle repatriation and legal status difficulties. Both countries then put in place legal frameworks to oversee the recruitment and employment of Indonesian migrant workers. The establishment of recruitment firms, the implementation of standard employment contracts, and the demand for work permits were all part of this.

Several concerns and challenges have arisen in relation to Indonesian migrant workers in Malaysia over the years. These include examples of exploitation, unfair treatment, unpaid salaries, passport confiscation, limited access to healthcare, social services, and work discrimination (Noor and Shaker, 2017). Human trafficking and forced work have also been reported. However, there is no doubt that the presence of Indonesian migrant workers has had a profound economic, social, and cultural impact on Malaysia. Indonesian workers contribute to Malaysia’s economy by filling labor vacancies in a variety of industries. Their presence has caused some Malaysians to be concerned about issues such as job rivalry, social integration, and cultural assimilation. To overcome the problem, the Indonesian and Malaysian governments signed a new MoU in Jakarta in August 2022 to establish a single-channel mechanism for all worker’s placement, monitoring, and return processes in Malaysia. The efforts include the introduction of hotlines and complaint channels, the legalization of undocumented workers, and increased labor management collaboration.

It is crucial to highlight that this is only a summary of the history of Indonesian migrant workers in Malaysia, which is a complicated and nuanced subject. Individual workers’ experiences differ, and both governments are making continual attempts to improve migrant workers’ working and living conditions. Thus, for some time now, the integration of migrant workers has been an important item on the political agendas of both countries, which are allegedly striving to strike a balance between their humanitarian responsibilities and their economic burdens. However, the predominant focus has been on the obstacles created by Indonesian migrant workers’ entrance, with considerably less attention paid to the potentially enormous benefits of their successful integration. They benefit not just the migrant workers themselves but also their host countries. They used to work as laborers in industries such as plantations, construction, agriculture, and services. As a result, they may be able to enter the labor market and contribute significantly to the Malaysian economy, easing host societies’ struggles with issues such as workforce shortages among local populations.

### 2.3 The model of intercultural communicative competence

It is commonly understood that ICC can assist individuals act in intercultural encounters, and various models focusing on this have been produced over the last three decades (e.g., Arasaratnam, 2016; Ladegaard, 2018). The most well-known of these models is that of Byram (1997, 2008) and Byram et al. (2001), which focuses not only on the language but also on the cultural aspects of communication, making it particularly relevant in intercultural contexts (Matsuo, 2014; Dalib et al., 2023). Byram’s ICC model is well-known for being pedagogically effective in formal language learning settings (e.g., Young and Sachdev, 2001; Chen, 2009), but also challenging models of language acquisition in which learners are evaluated using native speaker norms (e.g., Davies, 1991; Phillipson, 1992). Instead, it emphasizes the significance of communicating in a foreign language with people who have different cultural values, assumptions, and ideas. According to Byram (1997), what differentiates an intercultural speaker from a native speaker is their ability to establish relationships with others from different cultural backgrounds; their ability to “manage dysfunctions that arise in the course of interaction, drawing upon knowledge and skills”; and their ability to “establish a relationship between their own social identities and those of their interlocutor” (p. 38).

According to Byram’s model, there are five variables, sometimes known as savoirs, that are important in ICC. The first is attitude (savoir être), which is characterized as “curiosity and openness, readiness to suspend disbelief about other cultures and belief about one’s own” (Byram, 1997, p. 91). This requires the ability to perceive the world through the eyes of an outsider with different opinions and values. The second aspect is knowledge (savoir), which Byram (1997, p. 94) defined as “knowledge of social groups and their products and practices in one’s own and one’s interlocutor’s country, as well as knowledge of general processes of societal and individual interaction.” This mostly refers to understanding how social groupings and interaction processes work. The third aspect, interpreting and related skills (savoir comprendre), is defined as the “ability to interpret a document or event from another culture, explain it, and relate it to documents or events from one’s own” (Byram, 1997, p. 98). This emphasizes the necessity of learning the abilities required to acquire new knowledge and then integrating it with one’s prior knowledge. The fourth factor, discovery and interaction skills (savoir apprendre/faire), refers to the “ability to acquire new knowledge of a culture and cultural practices, as well as the ability to operate knowledge, attitudes, and skills under the constraints of real-time communication and interaction” (Byram, 1997, p. 98). This reveals the model’s true interactive nature. Finally, critical cultural awareness (savoir engager) is defined as “the ability to evaluate, critically and on the basis of explicit criteria, perspectives, practices, and products in one’s own and other cultures and countries” (Byram, 1997: 101). Ideally, this will lead to the acceptance of new ideas and values. Individuals who develop ICC – that is, intercultural speakers – can thus “effectively and appropriately mediate between world of origin and world of encountered difference” (Young and Sachdev, 2001, p. 83).

Byram’s ICC model, like most models, is not without criticism. Some scholars, for example, criticize it for not focusing solely on language components of communication, claiming that teaching “is best done through dialogue because culture is discourse” (Matsuo, 2014, p. 19). Most academics, on the other hand, value the model’s inclusion of a cultural component, particularly because “teachers in different classrooms in different parts of the world still ignore the importance of teaching culture as part of language study” (Gonen and Saglam, 2012, p. 29). Thus, Byram’s model is still frequently employed in foreign language didactics (see, for example, von Münchow, 2015; Tran and Seepho, 2016; Abdullah and Tandiana, 2019). While other models include cultural components, the ICC model was chosen as the best fit for the study. Because this research is co-orientational and regards “the ability of interlocutors to reach mutual understanding and a shared level of worldview as fundamental” (Spitzberg and Changnon, 2009; Critical Skills for Life and Work, 2021).

The concept of the intercultural speaker was extended to professional situations in the Critical Skills for Life and Work (CSLW) project, with a specific focus on migrant backgrounds, which emphasized the integration of individuals with migrant backgrounds into professional settings. These efforts aim to equip individuals with the skills necessary to navigate diverse workplace environments effectively (Bhugra and Gupta, 2010). This focus not only enhances the career prospects of individuals with migrant backgrounds but also contributes to fostering inclusive and culturally diverse workplaces, particularly in multicultural environments (Bhugra and Gupta, 2010). Corresponding to Byram’s five aforementioned factors, professional ICC refers to “key intercultural communicative skills, knowledge, attitudes, behaviors, and critical cultural awareness related to the process of successfully entering the professional sphere after a period of forced displacement” (Critical Skills for Life and Work, 2019, p. 6). Clear and effective communication with colleagues, clients, customers, and/or others is critical for everyone in a professional position. Because successful communication is built on “an ability to interact with others and collectively reach understandings” (Critical Skills for Life and Work, 2019, p. 6), migrant workers require at least some professional ICC to negotiate communicative encounters with cultural variations. Interlocutors must be able “to listen and show that they have understood, clarify meanings, repair breakdowns, and so on” (Critical Skills for Life and Work, 2019, p. 6).

It is important to note that integration, by definition, entails individuals retaining some degree of cultural integrity while also attempting to participate as an integral part of the larger society; thus, integration differs from assimilation, which entails individuals participating in the larger society at the expense of having to relinquish some degree of cultural integrity (Berry, 2011).

## 3 Research method

An exhaustive literature review of many academic publications to study the conceptualization and factors of ICC finds that they are not well established. As a result, qualitative interviews with migrant employees and their employers are done to guarantee that the concepts involved in this research fulfil the same function in organizations. Focus group discussions and face-to-face interviews were conducted in Malaysia in June 2023. The respondents are selected from Indonesian migrants’ workers (16 participants) and their employers (13 informants) from various sectors (i.e., services, plantations, construction, and domestic helpers).

Previous studies have supported the use of 12 participants or informants to achieve data saturation (Guest et al., 2006; Klaus and Nguyen, 2013). The focus group participants can be classified as skilled and unskilled Indonesian migrant workers in Malaysia. The study considers not only skilled workers—that is, those who have already successfully adapted to the workforce—but also unskilled workers—that is, those still in the process of transitioning into the Malaysian labor market (Table 1). While the employers are the experienced informants gathering information on ICC among the Indonesian migrant workers, most of them have dealt with Indonesian workers for more than 10 years and are entitled to provide a better reflection on their migrant worker’s ICC (Table 2).

**Table 1:** Profile of migrant workers.

Participants	Gender	Age	Education	Job sector
1	Male	Early Thirties	Upper Secondary	Cleaning Service
2	Female	Early Thirties	Degree	Cleaning Service
3	Female	Early Fifties	Upper Secondary	Cleaning Service
4	Female	Early Thirties	Upper Secondary	Restaurant (F&B)
5	Female	Mid-Forties	Upper Secondary	Cleaning Service
6	Female	Early Thirties	Primary	Cleaning Service
7	Male	Late Thirties	Upper Secondary	Construction
8	Female	Early Forties	Lower Secondary	Plantation
9	Male	Early Fifties	Lower Secondary	Restaurant (F&B)
10	Female	Late Thirties	Lower Secondary	Restaurant (F&B)
11	Female	Early Forties	Primary	Domestic Worker
12	Female	Mid-Fifties	Lower Secondary	Domestic Worker
13	Male	Late Thirties	Upper Secondary	Construction
14	Male	Early Forties	Upper Secondary	Welding
15	Male	Mid-Thirties	Islamic School	Construction
16	Male	Mid- Thirties	Upper Secondary	Construction

**Table 2:** Profile of employer.

Informants	Gender	Position	Age	Number of Indonesian migrant workers
1	Male	Manager	Early Forties	11–15
2	Female	Executive	Early Thirties	11–15
3	Male	Manager	Early Forties	11–15
4	Female	Executive	Early Thirties	11–15
5	Male	Director	Early Sixties	16–20
6	Male	Manager	Early Forties	56–60
7	Male	Supervisor	Late Twenties	56–60
8	Male	Supervisor	Early Thirties	56–60
9	Male	Director	Mid-Forties	26–30
10	Female	Assistant Manager	Late Twenties	26–30
11	Male	Supervisor	Late Thirties	26–30
12	Male	Manager	Early Fifties	11–15
13	Male	Executive	Early Thirties	11–15

They were used in the focus group and interview as participants and informants for three key reasons. For starters, they are a group of participants and informants with extensive expertise in intercultural communication who may represent Indonesian migrant workers and employers in Malaysia and provide information on many elements of the concepts in the study. Second, although drawn from a specific context, the participants and informants are highly literate and could effectively articulate the definitions and scopes of intercultural communication ability based on their own experiences. The information acquired will be in-depth, rich, and significant, facilitating the ICC’s meaning and strategy. Finally, migrant workers and their employers are two of the various stakeholders in the framework of intercultural communication. They are easily accessible and might be questioned further if necessary.

Throughout the data collection phase, the focus group discussion (FGD) and interview technique and procedures were used as recommended by Easterby-Smith et al. (2021). Participants of focus group and the informants (interview) were given ample time and space to respond to the questions posed. Because they live in Malaysia and are considered to be fluent in Malay, the focus group discussion and interviews were performed in both Malay and Indonesian. Due to company policy, interviewees requested that some of their personal information (e.g., names of interviewees and organizations) be kept confidential and strictly enforced at all times. For this study, the researchers obtained oral consent approval from the faculty’s Research and Publications Committee. An oral consent process is where the researcher and participant have a conversation to give information and obtain consent. There is no signed paper form. It is in accordance with the faculty’s research standard procedure that is normally used: (1) where literacy is a problem; (2) where there are cultural or political concerns with signing contracts like documents; and (3) where the existence of a paper record could put either the researcher or the participant at risk. Because the ethical committee determined that this study contained one or more of the aforementioned components, oral consent was obtained for the purpose of this study.

The data analysis procedure begins with transcribing the audio data from the focus group discussion and interview into text. The text was then analyzed using the QSR Nvivo Version 12.0 qualitative analysis software. The use of Nvivo simplifies data analysis and makes it more dependable, accurate, and clear (Gibbs, 2002). Deductive content analysis is used to categorize qualitative data based on key terms or themes (Krippendorff, 1980; Hinkin, 1995). Given that certain new elements and themes emerged during the research, inductive content analysis was modified to some extent. Various ICC factors surfaced as a result of the coding. Following that, the major findings on the factors of intercultural communicative competence (ICC) in the integration of Indonesian migrant workers into the Malaysian labor market are provided.

### 3.1 Participants

While the majority of the Indonesian laborers came from a variety of occupational backgrounds, most of them had a basic understanding of Malaysian culture prior to their arrival. They could all have rudimentary discussions and have basic competency. The competence of Indonesian migrant workers was substantially higher than that of other migrant employees (Bangladeshi, Nepalese, and Vietnamese), as would be expected.

### 3.2 Methods and materials

Firstly, focus groups were used to obtain data from migrant workers because they allowed participants to share their collective ideas and practices. They are more likely to elicit natural and spontaneous responses (Callejo, 2001), providing insight into salient social representations in communicative conversational contexts (Iglesias-Álvarez and Ramallo, 2003). Furthermore, focus groups are very beneficial for exploratory studies of new study fields, such as ICC among migrant workers (Hornsby, 2022). Some scholars say that only larger focus groups offer data that accurately reflects the participants’ aggregate perspectives; others contend that focus groups can be “small or large” (Kamberelis and Dimitriadis, 2010). Multiple studies on Indonesian migrant workers found that focus groups with female and male participants from various industries were more helpful than a single, larger focus group. As the results show, there were some contextual differences that confirmed our decision. In addition to Byram’s ICC model, participants were asked open-ended questions regarding their perceptions of Malaysian life, the learning environment in Malaysia, their employment experiences in Indonesia and Malaysia, and their life dreams, expectations, and goals.

Second, semi-structured interviews were also utilized to collect information from employers using Byram’s ICC model, which included attitude, knowledge, skills, interaction, and cultural awareness. Because there were fewer participants in the employer group and their busy schedules, this method was seen as more acceptable than a focus group. Interviews cannot provide insights into communal beliefs and practices that focus groups may. Interviews, on the other hand, like focus groups, tend to bring to the surface both competing thoughts as well as commonalities and shared understandings among employers, resulting in rich and in-depth data (Karatsareas, 2022). Furthermore, interviews are especially useful among Malaysian employers because they generate a different perception, allowing them to set the research agenda and lead the discourse toward themes that they believe are important (Edly and Litosseliti, 2010; Karatsareas, 2022).

The employer was asked open-ended questions about their experiences with Indonesian migrant workers, their knowledge of culture, language learning, and linguistic adjustment while communicating with the migrant workers, their education and training experiences that helped them communicate well with their migrant workers, the factors that helped their migrant workers adapt to the local culture, the factors that were a particular hindrance, and the key advice.

### 3.3 Procedure

The invitation to participate was sent to migrant workers in partnership with Indonesian Migrant Workers (PMI) in Malaysia. PMI is an active association for Indonesian migrant workers in Malaysia. They were active in a variety of activities for migrant workers’ welfare, including education, social services, and training. Sixteen migrant employees from various sectors have agreed to engage in the focus group discussion through PMI. The focus group discussion session lasted 126 min and employed pre-prepared questions and an interview methodology. The focus group discussion has been set up as a roundtable discussion by the partner university in Kuala Lumpur. The participant attended physically, and the session followed the focus group protocol. The focus group discussion was conducted in Malay, and they were moderated by three researchers (one from Malaysia and two from Indonesia).

While, 13 employers were interviewed to supplement the data from migrant employees. As a minimum, firms should have hired at least five Indonesian migrant employees. In Malaysia, the type of organization should fall under the category of small and medium enterprises (SME). SME sales turnover of RM50 million or full-time employees of no more than 200 workers; and in services and other industries, sales turnover of no more than RM20 million or full-time employees of no more than 75 workers (Bank Negara Malaysia). The SME has been chosen because employers have direct communication with their migrant workers and are involved in daily communication and interaction in the workplace. It will give a better input on the professional ICC of the migrant worker.

Face-to-face interviews with employers were done in Klang Valley (Kuala Lumpur, Putrajaya, Petaling Jaya, and Shah Alam) due to geographical distance and time restrictions. In interviews, the same pre-prepared questions and interview protocol particular to the companies were employed. Their average running time was 40 min. All data collection from focus group and interview sessions were videotaped and transcribed, and the Indonesian and Malay languages were translated into English.

### 3.4 Data analysis

We employed thematic analysis on both focus group and interview data (e.g., Braun and Clarke, 2006, 2012). We began by categorizing the data using Byram’s five ICC factors: (i) attitudes; (ii) knowledge; (iii) interpreting and related abilities; (iv) discovery and interaction skills; and (v) critical cultural awareness. The data was then inductively examined to seek for repeated subjects and patterns within each of the five core themes.

## 4 Result and discussion

The findings of this study were separated into two section and then the discussion and conclusion were made based on the migrant’s workers and their employers feedback.

### 4.1 Employee

According to the focus group findings, at least some of the migrant employees had established some professional ICC in terms of attitudes. The Indonesian migrant workers had accepted that people in Malaysia are similar to people in their home country, but co-workers from Bangladesh, Vietnam, and Nepal are more difficult to grasp. They also learned that the multi-racial country of Malaysia required them to be aware of other races, such as Indians, Chinese, and Bumiputra from Sabah and Sarawak (East Malaysia). This can be seen as their emerging ability to observe Malaysian mixed cultures through the eyes of an outsider with a distinct set of values and beliefs. According to participant #3*, “working in Malaysia opened my eyes to Malaysian culture. I’ve worked for Malay and Chinese employers, and their approaches are really different.”* In terms of attitudes, there was little evidence that migrant employees had formed professional ICCs.

In terms of attitude, it is very important for the migrant workers to align with a local culture, and they have to understand exactly what is going on in their new environment. One of the participants in the focus group [participant #7] added that an open attitude is important by saying that “*I try to learn local culture and language in order for me to communicate well with my local co-workers and employers*.” This statement was also supported by [participant #1], who highlighted his different religion with the majority of the community. An attitude of respect and openness is vital for the migrant worker. He said *“I work in the northern part of Peninsular Malaysia, where the majority of the people are Muslims, and my attitude needs to be more open and adapt to them.”* Such discourse suggests that the attitude factors of ICC capture key aspects of the construct and concept of ICC.

Besides their attitude toward a local culture, the migrant workers need to adapt by mingle around with the locals. For example, the migrant workers should be involved in community activities such as weddings, religious gatherings, and social gatherings. One of the participants [participants #4] mentioned that the locals will be more welcome and acceptable for those who can dilute with their culture. He said, *“I involved in community activities, just as I have done in Indonesia. The similarity of cultures makes it easy to communicate with and understand them.”* When discussing the professional ICC, most of the participants have no problem communicating with and understanding their employers. One of the participants [participant #12] responded, *“Maybe we have a similar culture, and the language barriers are not the major problem. Sometimes, we talk beyond the works, such as family, food, and our social life.”* Due to the similarity, the Indonesian migrant workers feel at home and comfortable adapting to the local culture, and they can stay in Malaysia for a long time.

The second factor of ICC is knowledge. The findings suggest that the knowledge factors capture and reflect the ICC among Indonesian migrant workers appropriately. Evidently, the majority of participants largely agreed with the factors. Knowledge of the host society’s language, values, and culture is not enough; interlocutors must also be able to listen and demonstrate understanding, clarify meanings, and mend breakdowns, among other things. The participants agreed that knowledge about the host country is vital for their survival. One of the participants [participant #5] responded: *“After more than 10 years of living here, my knowledge about Malaysia is deeper. I can communicate well with the different races and even the local dialect of the Malay community. For those who come to Malaysia as tourists, they may face difficulty understanding..”* Another participant [participant #15], who works with a Chinese employer, stressed her knowledge of Malaysia as a multicultural society. She said, *“My boss is Chinese, and his customer majority is Malay, and I live in a mixed Malaysian society. Therefore, I should be knowledgeable and know how to adapt to this situation.”* Knowledge about the language, customs, values, and traditions of the local people is a must for the Indonesian migrant workers.

When discussing the working environment, the participants mentioned the differences among employers. According to participant #10, *“Chinese employers are concerned with outcomes and productivity, while Malay bosses are concerned with outcomes and relations. Both of them are appreciated as fast learners and knowledgeable workers.”* They believe both workers and employers are dependent on each other. Employers need a good worker for their business survival, while migrant workers are seeking a good place to work for their lives. The participant [participant #16] mentioned: *“My employers are very kind to me because of my knowledge, and they are very dependent on me.”*

Participants in the focus groups revealed indications of relating and interpreting skills. The participants demonstrated evidence of this component in terms of the ICC in particular. The religion aspect was the most relevant topic of discussion in this regard, given Malaysia is an Islamic country. The female participants reflected that shaking hands with men is seen as uncommon by most Muslim people in Malaysia due to religious reasons. One of the female participants [participant #6] mentioned that *“after working a few years in Malaysia, I understand the behavior of the Muslim community and have started to practice the same. I do not shake hands with a man.”* This is supported by one of the participants [participant #4] who is working in the agriculture sector: *“I did not wear the hijab when I started working in Malaysia, but when I see the majority of Muslim females wearing the hijab, it motivates me to wear it.”*

While working in Malaysia, most of the participants are able to interpret such cultures and know the reasons behind them. The participant thinks that migrant workers need to adapt to Malaysian habits: *“That is Malaysian culture. We come to Malaysia, so we need to follow their culture. We also need to understand that a little”* [participant #14]. Another participant says that in Indonesia, too, some women do not wish to shake hands with men. She elaborates, *“Indonesia is a big country with a more diverse culture than Malaysia. Some cultures in Indonesia are quite similar to Malaysia, so we have no problem adapting such behavior”* [participant #16]. These findings show that certain Indonesian migrant workers were at various levels of developing ICC in terms of understanding and related abilities, but after a few years they were able to integrate into Malaysian culture. These findings show the importance of ICC training for migrant workers before they travel to Malaysia to work. This will help them understand the general knowledge of the ICC.

The focus group data analysis show that the Indonesian migrant workers had gained a significant level of ICC in terms of exploration and interpersonal skills. They had not only learned about Malaysian culture, but they were also able to reflect on it from their current position. Migrant workers have claimed that acquiring and understanding the host country’s language is critical for successful integration and participation in the labor force. According to them, learning the language and using it in daily life has improved their Malay ability. One of the female participants commented, *“Even though Malaysia and Indonesia have similarities in language, the standard Malay is necessary to learn. Some Indonesian words have a different meaning in Malay. Furthermore, Malay is widely spoken, and it is important to communicate with Chinese or Indians. They sometimes have difficulty understanding [participant #6].”* In the same vein, another participant supported this opinion by saying, “*It is highly appreciated when you speak Malay, and at the same time, it will appeal more to the host if you speak their language* [participant #8].” The other participant [participant #13] added: *“It is not just a matter of the language; it is the culture that you are aware of. When you learn the Malay language, you also learn the Malaysian culture. If you learn the dos and don’ts of the people, then it will make you comfortable and confident to interact with the locals.”*

Indonesian migrant workers got the opportunity to reflect on what else had aided their integration in Malaysia. The participant emphasized the importance of being truly interested in and open to other people and new innovations. They also discussed the benefits of developing a strong network and trust. For example, one male participant noted: *“I have worked with the same employer for more than 15 years. My relationship with him is based on trust, and I tried to deliver the best I could for my job [participant #9].”* The participant also saw the attitude toward work as a crucial aspect of integration in the Malaysian workplace. Participant #14, for instance, mentioned that the *“employers want the task given to be done accordingly. I, as a worker, follow the instructions and do as good as I can.”* Participant #5 also offered her an insight about Malaysian culture: *“With the interaction with the co-workers and local people, I discovered many things about Malaysia. I always asked my Malaysian friend about the sensitive issues in Malaysia, so that it was helpful for me to learn how to behave.”*

Importantly, the participants displayed their ability to reflect on the issues they faced, especially the obstacles posed by Malaysia’s bureaucracy and system. They stated that seeing such hurdles as opportunities was the key to their successful integration into the Malaysian work market. They agreed that the migrant workers needed to follow Malaysian law and regulations. However, there are also cases where migrant workers are not treated humanely. For example, participant #16 stated explicitly that *“there are cases where the employer mistreats their migrant workers. Although the cases are small as compared to the total number of migrant workers, they need to be taken seriously.”* Participant #7 advised that *“those who wish to work in Malaysia should come through the official procedure. At least our welfare is taken care of, and we can report to the authorities if anything happens to us.”*

More evidence of critical cultural knowledge was displayed by the migrant employees. In the focus groups, for example, everyone commented on the qualities of Malaysian employers, categorizing them as distinct based on race. For example, participant #7 explained: *“When we talk to a Chinese employer, sometimes they have a problem understanding, so I will use a standard Malay language. But it is also the same for the Malay bosses, who always use their own state dialect, such as Kedah and Kelantan.”* The participant noticed that Chinese employers are too focused on work and always concerned about the outcomes. Participant #5 explained: *“My employer gives me a new task even though my current job is still incomplete.”*

The migrant employees were also able to critically remark on and compare particular customs in their host country to those in their home country. For example, one participant stated that he thought it was excellent that all workers in Malaysia were treated equally. He said, *“My boss is always fair to everyone, and he treats me the same as local workers”* [participant #10]. This was far more formal to him than the working atmosphere in his home country.

### 4.2 Employer

According to the interview data, an employer believes that Indonesian migrant workers have a significant amount of professional ICC in terms of their views. Employers stated willingness to accept that things are done better for Indonesian migrant workers than for other workers. One of the employers [Informant #3] mentioned, *“We have fewer problems with Indonesian migrant workers. Besides hard work, they are respectful, polite, and always follow the instructions given. It’s easy for us to work together.”*

The cultural similarity between Indonesia and Malaysia makes employers more comfortable dealing with Indonesian migrant workers. For instance, one of the employers recommended that language is another added value for Indonesian migrant workers in Malaysia. According to informant #5, *“we understand each other well. Maybe a few words are different, but I understand the whole context while communicating with them.”* Informant #2 also added, *“I learn to talk to them using their language. We need to complement each other because interaction requires compromise. By doing that, as an employer, I believe it will create a good working environment for them.”* However, another employer [informant #13] has a different opinion on the language: *“I communicate in Malay with my Indonesian migrant workers. They should learn the local language in order to survive here. So far, they are very open and have no problem communicating with me.”* The statement illustrates the attitude of Indonesian migrant workers who are willing to adapt to the host culture, and some employers have also tried to adapt to the culture of their migrant workers.

One of the employers [informant #5] emphasized the importance of migrating workers having the correct mind-set and thinking about their strengths and professional goals in the host country. He mentioned, *“The Indonesian workers are very hardworking and willing to learn about new things. They should take advantage of the similarity culture to gain social experiences other than working experience.”* Because of the good attitude and fewer cultural barriers, the majority of employers agreed that they are more comfortable with Indonesian migrant workers. One senior employer that had been hiring Indonesian workers for 30 years mentioned: *“Last time I traveled to Banyuwangi to recruit a worker for my company. They are hardworking and polite. As a boss, it makes it easy for me to deal with”* [informant #5]. Another employer also stressed the same opinion: *“I hired a worker from Madura, and 80 percent of my migrant workers are from there. Most of them are relatives and come from the same village. I also appointed a supervisor from Madura to make it easy for me to handle my workers”* [informant #10]. Employers feel that having an open approach will help them integrate faster, participate in the Malaysian labor market, and become acquainted with their new surroundings.

The employers confirmed the knowledge of Indonesian migrant workers about intercultural communication in Malaysia is better than others (e.g., Nepalese, Bangladeshi, and Vietnamese). According to the interview data from the employers, the Indonesian migrant workers had also formed an understanding of how social groupings and identities function in Malaysia. This ability to reflect on certain talents and qualities that facilitate integration in Malaysia. The informant #12 elaborated: *“The cultural knowledge among the Indonesian migrant workers is good in general, but some workers from provinces such Flores and Papua find it quite challenging to adapt to the host culture.”* This is evidenced by their comments targeted at assisting others in their integration journey. For example, one of the male employers indicated that understanding of the Malaysian labor market is useful in obtaining employment in Malaysia. He said, *“The Malaysian government opened a few sectors (e.g., plantations, construction, agriculture, services, and domestic helpers). Therefore, the migrant workers should have knowledge of the expectations of employers while working with them.”* [informant #9]. He stated that social qualities such as the capacity to manage the employer’s expectations were the most beneficial to migrant workers. This statement was supported by informant #8, who mentioned that *“work experience and education have been particularly helpful in migrant workers’ integration into the Malaysian workforce.”* He also spoke about the significance of cultural awareness and linguistic skills: *“the cultural knowledge will help them in the integration process and add value to their social skills; so the more opportunities they have.”*

Overall, our data provide minimal evidence of employers’ perceptions of interpretive and relational abilities among Indonesian migrant workers. However, the fact that there is some evidence among the Indonesian migrant workers could be seen to indicate further progress in their development. For instance, one of the employers mentioned: *“At the beginning, the Indonesian workers had a bit of a problem understanding the instructions in Malay. Therefore, I recruit the supervisor among them who is more senior to guide. But in less than a year, they can integrate the work environment in Malaysia”* [informant #7]. It is not a problem for them to comprehend features of Malaysian culture and relate them to the culture of their country of origin. In an interview with Malaysian employers, for example, the informants discussed the effects of cultural differences while attempting to integrate into the Malaysian labor market, noting that they were not a hindrance. Due to the similarity, the employer can understand the behavior of Indonesian migrant workers. They demonstrated this by showing how the modesty of the Indonesian migrant worker was highly guarded, with the reason being the taboo connected with self-praise in his place of origin. For example, one of the employers noted that *“my workers are very polite when talking to me. This behavior is similar to the Malay culture, which always talks politely to the elder and the superior. But sometimes I advised them to be straightforward because the work culture is different from the social culture”* [informant #9]. Indonesian migrant workers should be aware that self-praise is required to succeed in the Malaysian labor market, particularly in the workplace and throughout one’s career. However, informant #9, who has experience with many Indonesian workers, believes that this kind of skill depends on their education level and the specific culture of their place in Indonesia. He said, “The Indonesian workers can be classified based on their province in Indonesia. From my experiences, the Javanese are polite workers.” Most of the employers believe that migrant workers from Indonesia have an advantage working in Malaysia due to similarities in language, culture, and religion.

The interviews with the employer’s data show that Indonesian migrant employees gained some knowledge of Malaysian culture and were able to reflect on it (discovery and engagement). This, according to the employers, is demonstrated by the worker’s knowledge that they would have to overcome hurdles in order to go where they wanted to go. For example, informant #12 realized that his workers have no problem with them, and he commented on this by saying, *“My workers learn to understand the Malaysian culture throughout their employment. They will refer to me if they are unsure about something.”* The other employer repeated that the *“similarity of language and culture is an advantage for Indonesian migrant workers in Malaysia. We cook and eat the same food, so they have less obstacles to integrate with the locals”* [informant #10].

Employers were also conscious of the significance of knowing the language of the host country. The majority of employers feel that having adequate language abilities will make it easier for them to expand their social networks, which will eventually help them live a pleasant existence like a home. They all agreed that language and cultural knowledge are crucial for migrant workers’ integration in Malaysia. As informant #9 mentioned, *“The faster they can dilute with local culture, the faster they can be accepted by the locals.”*

Employers also said their Indonesian employees’ ICC in terms of discovery and interaction abilities was good. They would do well and achieve their professional goals if they had assistance. For instance, all employers agreed that the migrant workers would need to attend basic training on language and culture before entering the Malaysian labor market. One of the employers mentioned, *“It is a good idea to have basic training and the employer’s expectations among the Indonesian migrant workers. It will be a ‘soft landing’ for workers when entering the new work environment and also minimize the cultural shock and retention”* [informant #2].

The employer’s interview data shows minimal indication of migrant workers’ critical cultural knowledge. The only relevant topic they covered was the contrasts in working cultures between Indonesia and Malaysia. For example, informant #1 mentioned: *“On the first day they work with my company, I stress over the working culture in Malaysia. They should know my expectations and how to get the job done.”* In the same vein, one of the informants, #12, explained: *“Of course there are different ways to work in an international environment; the approach, salary, and communication style are also different.”* Informant #4 added, *“The Malaysian working culture is different, especially the culture of the private organization. The worker should be aware of the employer’s expectations, particularly productivity, and focus on outcomes.”* Overall, the employers believed that their migrant workers were a bit critical, but they tried not to openly criticize because they did not want to create conflict.

### 4.3 Discussion

The interpretation of our findings is reinforced by earlier research that revealed the importance of ICC in a variety of other sorts of intercultural interactions. Other factors, such as the fact that the Indonesian migrant workers had advanced language skills due to a commonality, got Malay degrees, and built networks in Malaysia, are likely to have played a role. This study supports Shenkar’s (2001) claim that when there is less cultural barrier between one’s culture of origin and the new host culture, the situation is less stressful. However, all of these things would have indirectly contributed to the individuals’ ICC. Overall, the outcomes of our study suggest that increasing ICC may be advantageous for Indonesian integration into the job market of the host country.

Our findings indicate that expanding the ICC model to incorporate similarities in addition to the other five parameters may be beneficial. The Indonesian migrant workers in our study shown comparable signals of resilience to those in Ganassin and Young’s (2020) study: they kept a positive outlook in the face of adversity, and they appeared motivated to perceive impediments as challenges to be conquered. Indonesian migrant workers, on the whole, appear to be robust, as seen by their journey. Nonetheless, our data indicates that Indonesian migrant workers’ resilience was far advanced.

A closer examination of the data reveals one factor that stands out from the rest: Indonesian migrant workers were more advanced than other migrant workers in terms of attitudes, knowledge, interpreting and relating skills, and discovery and interaction skills; however, the data from Indonesian migrants contains less evidence of critical cultural awareness. More research would be required to confirm this. Notably, the data indicate that the development of ICC was not at the same stage for all components and that there were disparities across participants within the factors as well. This is demonstrated by the migrant worker’s ability to interpret and relate. However, the perspectives of Indonesian migrant workers differed, with some presenting themselves at different stages of developing ICC in terms of interpreting and relating skills, but they showed an open attitude to assimilate with local cultures in order to fully participate in the host country.

Many of the problems and impediments to employment for Indonesian migrant workers in our study differed from those outlined in studies on refugees conducted in the Netherlands and the United Kingdom (Ganassin and Young, 2020; Schukking and Kircher, 2022). The similarities may be on the topic of dealing with bureaucracy, but participants do not cite weak language skills or a lack of networks as important issues. Furthermore, our findings support the arguments of Ganassin and Young (2020) and Schukking and Kircher (2022), who argue that the ICC model needs to be further developed to fully accommodate migrant workers’ intercultural encounters because, as stated in the introduction, their circumstances and experiences differ significantly from those of other migrants and individuals simply traveling abroad. The traditional ICC model does not account for this, but Byram (2009) concedes that his model’s list of elements is not exhaustive. Thus, Ganassin and Young (2020) advise that resilience, defined as “the capacity of individuals to cope successfully with significant change, adversity, or risk” (Lee and Cranford, 2008, p. 213), be addressed while investigating ICC among migrant workers.

As a result, it would thus make sense to incorporate ICC training into the modules of the integration courses as well as the language for new Indonesian migrant workers seeking to enter the Malaysian workforce. For instance, the Indonesian Migrant Workers Protection Agency (BP2MI), which has responsibilities to protect Indonesian migrant workers during pre-placement, placement, and post-placement, should integrate intercultural communication competency training alongside other professional skills. It would also be beneficial to provide ICC awareness training to agency professionals and job consultants whose work involves assisting migrant workers (Akanmu et al., 2023). As of April 30, 2023, there are 362 agencies registered under the Indonesian Migrant Workers Placement Agency (P3MI) who have obtained a written permit from the Ministry of Manpower to operate the placement service for Indonesian migrant workers. Furthermore, if ICC training is provided to migrant workers and professionals who assist them, it should also be provided to host-country employers who contact migrant workers. The Ministry of Human Resources Malaysia can collaborate with a Private Employment Agency to provide short ICC training to companies applying for the employment of foreign workers. Arguably, it would also be advantageous if members of the host country in general gained a certain degree of ICC in order to aid the assimilation of newcomers. To begin addressing this, ICC training among members of the host country could be good. While much more needs to be done, we argue that explicit teaching of ICC to migrant workers themselves, as well as increased awareness of the importance of ICC among migrant workers who are supposed to help them, can both be considered reasonable first steps toward assisting and promoting migrants’ labor market integration. Clearly, this can only succeed if the teaching and awareness-raising of ICC skills is done correctly, with the goal of actually assisting migrant workers and the possibility of tactics to finally bring about wider structural changes.

As previously stated, additional study is required to determine whether our findings apply to highly educated migrant workers (expatriates) in Malaysia. However, if this is true, our findings have significant implications for increasing the integration of these groups into the Malaysian job market. The findings imply that increasing ICC (including resilience) is good for highly educated migrant workers’ integration into the host society’s labor market because it helps them deal with the multiple problems and impediments they face during this process. Furthermore, this feature of our findings emphasizes the need for additional research, particularly with regard to the various gender groupings among migrant workers. Gender disparities in the labor market exist in both Indonesia and Malaysia (World Economic Forum, 2021), as seen by fewer full-time jobs and lower economic involvement among women. As a result, it is not surprising that women experience labor market integration differently than men. More research is required to determine whether this pattern is systematic and if it also applies to Indonesian workers.

The study also found that the majority of ICC research has focused on applying Western theories and practices to the Asian environment. The applicability of ideas utilized across nations is one of the primary difficulties (Tsui, 2006). Today, as many scholars have argued, the literature and academic community require new contexts of study in order to create external validity (Boyacigiller and Adler, 1991; Peng et al., 1991). Because most Western theories are built on a set of assumptions related to their culture and institutional underpinnings, fresh research is necessary to respond to the global examination by examining the link of current theory to the new setting (Tsui, 2006). As a result, investigating the ICC model among Indonesian migrant workers in Malaysia added to the existing literature because Asian cultural backgrounds differ significantly from those of Western countries (Hofstede, 1980; Abdullah and Lim, 2001). As a result, the applicability of Byram’s (1997) model has been expanded into a new environment in these studies.

Furthermore, the notion of the Western context’s individualistic culture may not apply in the new setting (Indonesia and Malaysia), which is culturally more collectivist (Hofstede, 1980). As a result, the study’s findings show that any application of Western theory must be contextualized when applied to an analysis of a similarly related issue in Malaysia. In addition, not all of the ICC factors identified in the literature are applicable to the Malaysian setting. However, our study demonstrated that the same criteria can be employed in other circumstances as well. Moreover, the important implications of the current research are the establishment of ICC factors among the Indonesian migrant workers, and it was beneficial for policymakers and employers to understand the migrant worker’s needs. This will assist them in tailoring the best practices of the ICC that are suitable for the Malaysian labor market.

## 5 Conclusion

The work presented here definitely has limitations. For example, the migrant worker’s reflections may have been influenced by their circumstances at the time of data collection. When repeating one’s experiences and progressing from a position of success, it may be easier to assess the worth of ICC in forging one’s integration into the Malaysian job market. Furthermore, there are concerns with the ICC study because it is impossible to assess the important components; all we could do was conduct an exploratory comparison of the migrant workers and their employers, thereby establishing supplementary data.

The non-representative nature of the participant sample as a result of the sampling process, as well as the small sample size among participants in the focus group discussion, are also significant limitations. Furthermore, it would have been excellent to have a participant sample that included additional sectors and was national in scope. As a result, we cannot generalize the findings of our study to all migrant workers in Malaysia, and we clearly accept that any conclusions we might draw are only preliminary and limited to Indonesian migrant workers.

To confirm our findings, more study with larger, random, and multi-sector samples is required. Nonetheless, the findings of this first exploratory research of Indonesian migrant workers and their employers in Malaysia are significant. The data show that the ICC of Indonesian migrant workers was comparatively more advanced than that of other migrant worker groups, such as Nepalese, Vietnamese, or Bangladeshi. Thus, the findings provide a preliminary answer to our research question by implying that the ICC model developed by Byram (1997) may have played an important role in the migrant worker’s adaptation process of overcoming the numerous challenges and barriers to their integration in the host society’s labor market.

In conclusion, this study was conducted based on two main gaps. First, the specific factors of ICC among Indonesian migrant workers are poorly explored, and there is only a limited literature review available. Second, ICC strategies have different views from the Indonesian migrant workers due to cultural and language similarities, which makes the degree of ICC factors diverse from other groups of migrant workers. As a result, the academic contribution to this research is an examination of ICC among Indonesian migrant workers in Malaysia, with the goal of supporting policymakers, job seekers, employers, and job agencies in understanding the primary variables of intercultural communication competence.

## Data availability statement

The raw data supporting the conclusions of this article will be made available by the authors, without undue reservation.

## Ethics statement

The studies involving human participants were approved by the Ethics and Publication Committee of Sebelas Maret University. The research was conducted in accordance with national legislation and institutional requirements. The participants provided oral informed consent for participation in the study. The Ethics Committee waived the requirement of written informed consent.

## Author contributions

Djatmika: Conceptualization, Data curation, Formal analysis, Funding acquisition, Investigation, Methodology, Project administration, Resources, Supervision, Writing – original draft, Writing – review & editing. BM: Conceptualization, Data curation, Formal analysis, Investigation, Methodology, Project administration, Resources, Software, Supervision, Validation, Visualization, Writing – original draft, Writing – review & editing. RS: Conceptualization, Data curation, Funding acquisition, Methodology, Project administration, Resources, Writing – original draft, Writing – review & editing. AW: Conceptualization, Formal analysis, Funding acquisition, Investigation, Methodology, Project administration, Resources, Writing – original draft.
